# Risk factors for bloodstream infections and mortality in carbapenem-resistant versus susceptible *Klebsiella pneumoniae*: a six-year retrospective cohort study

**DOI:** 10.1186/s12879-026-12960-0

**Published:** 2026-03-02

**Authors:** Debao Li, Keyang Li, Hongliang Dong, Dandan Gong, Chen Shen, Yiqi Pei, Dongmei Ren, Siwei Yang, Zhizhong Lu, Nana Wu, Zhenyao Wei, Junmin Li, Wenjuan Yan, Yi Li

**Affiliations:** 1 Department of Clinical Laboratory, Jiaozuo People’s Hospital, Jiaozuo, Henan China; 2 Department of Clinical Pharmacy, Jiaozuo People’s Hospital, Jiaozuo, Henan China; 3 Department of Gynecology, Jiaozuo People’s Hospital, Jiaozuo, Henan China; 4 Department of Medical Oncology, Jiaozuo People’s Hospital, Jiaozuo, Henan China; 5 Department of Clinical Laboratory, Henan Provincial People’s Hospital, Weiwu Road 7#, Jinshui District, Zhengzhou, Henan 450003 China

**Keywords:** Bloodstream infections, Carbapenem-resistant *Klebsiella pneumoniae*, Risk factors for CRKP-BSI, Mortality rate

## Abstract

**Background:**

Carbapenem-resistant *Klebsiella pneumoniae* (CRKP) is a critical global health threat, with rising resistance rates and high mortality in bloodstream infections (BSIs). In China, the prevalence of CRKP has increased, particularly in Henan Province, where resistance rates are among the highest. However, regional epidemiological data are limited. In the present study, CRKP-BSI and carbapenem-susceptible *Klebsiella pneumoniae* BSIs (CSKP-BSI) were investigated, resistance patterns were analyzed, and risk factors for CRKP-BSI and KP-BSI were evaluated. The primary objective was to identify the risk factors for CRKP-BSI, while the secondary objective was to determine the risk factors for in-hospital mortality among patients with KP-BSI.

**Methods:**

The present study included patients with KP-BSI who were hospitalized in a tertiary hospital in Henan with 1,800 beds from January 1, 2019, to December 31, 2024. A retrospective analysis of patients with KP-BSI was conducted, and clinical data on patient demographics, comorbidities, and discharge outcomes were collected. Univariate and multivariate regression analyses were performed to identify independent risk factors for CRKP-BSI and in-hospital mortality rates associated with KP-BSI.

**Results:**

A total of 414 patients with KP-BSI were included in the study, comprising 90 patients with CRKP-BSI and 324 patients with CSKP-BSI. The independent risk factors for CRKP-BSI included a history of intensive care unit (ICU) admission (OR = 10.49, *p* = 0.0002), hospital-acquired infection (OR = 9.34, *P* < 0.0001), hypertension (OR = 2.85, *P* = 0.0106), and surgery within 30 days prior to infection (OR = 5.88, *P* = 0.0002). The overall in-hospital mortality rate was 11.8%. The mortality rates of patients with CRKP-BSI and CSKP-BSI were 21.1% and 9.3%, respectively. The independent risk factors for in-hospital mortality due to KP-BSI were a history of ICU admission (OR = 6.03, *P* < 0.0001) and the presence of lower respiratory tract infections (OR = 2.44, *P* = 0.0103).

**Conclusions:**

In this comprehensive study, we identified several critical risk factors for CRKP-BSI, including ICU admission, healthcare-associated infection, hypertension, and recent surgical intervention. Moreover, ICU admission and lower respiratory tract infection were identified as independent predictors of in-hospital mortality in patients with KP-BSI.

**Clinical trial number:**

Not applicable.

## Background

Carbapenem-resistant *Klebsiella pneumoniae* (CRKP) has emerged as a critical global public health threat because of its widespread dissemination [1, 2]. For 15 years, there has been a consistent association between CRKP-induced bloodstream infections (BSIs) and elevated mortality rates among hospitalized patients worldwide [3]. The China Antimicrobial Resistance Surveillance Network (CHINET), a nationwide surveillance system initiated in 2004 that currently includes 78 member hospitals, reported a substantial increase in carbapenem resistance among *Klebsiella pneumoniae* (KP) isolates in 2023 [4]. Specifically, resistance to imipenem increased from 3.0% in 2005 to 24.8% in 2023, and resistance to meropenem increased from 2.9% to 26.0% during the same period. The 2023 National Antimicrobial Resistance Surveillance Report indicated that the resistance rate of *KP* to carbapenems in Henan Province was 19.9%, representing the third highest prevalence among all monitored regions in China [5]. A multicenter cross-sectional study focusing on ICU patients in Henan demonstrated a 20.9% CRKP colonization rate, with the Jiaozuo region showing particularly high endemicity [6].

Şimşek and Butt [7] reported that independent risk factors for CRKP-BSI included a history of seismic events and the use of carbapenem antibiotics. Conversely, a study conducted at a tertiary hospital in North China identified independent risk factors for CRKP-BSI, including blood purification (continuous renal replacement therapy [CRRT], bedside hemofiltration, hemodialysis, and plasmapheresis), bronchoscopy, surgical procedures, and the use of carbapenem antibiotics and tigecycline [8]. Research has been conducted on independent risk factors for CRKP-BSI, concluding that heterogeneity exists. This study aimed to evaluate the resistance rates of KP-BSI in this region, identify the risk factors associated with CRKP-BSI, and assess the risk factors for inpatient mortality due to KP-BSI. In light of the global threat posed by CRKP, this study is anticipated to facilitate the acquisition of invaluable insights into the control of CRKP infections and enhancement of clinical outcomes.

## Materials and methods

### Study design

**Fig. 1:**
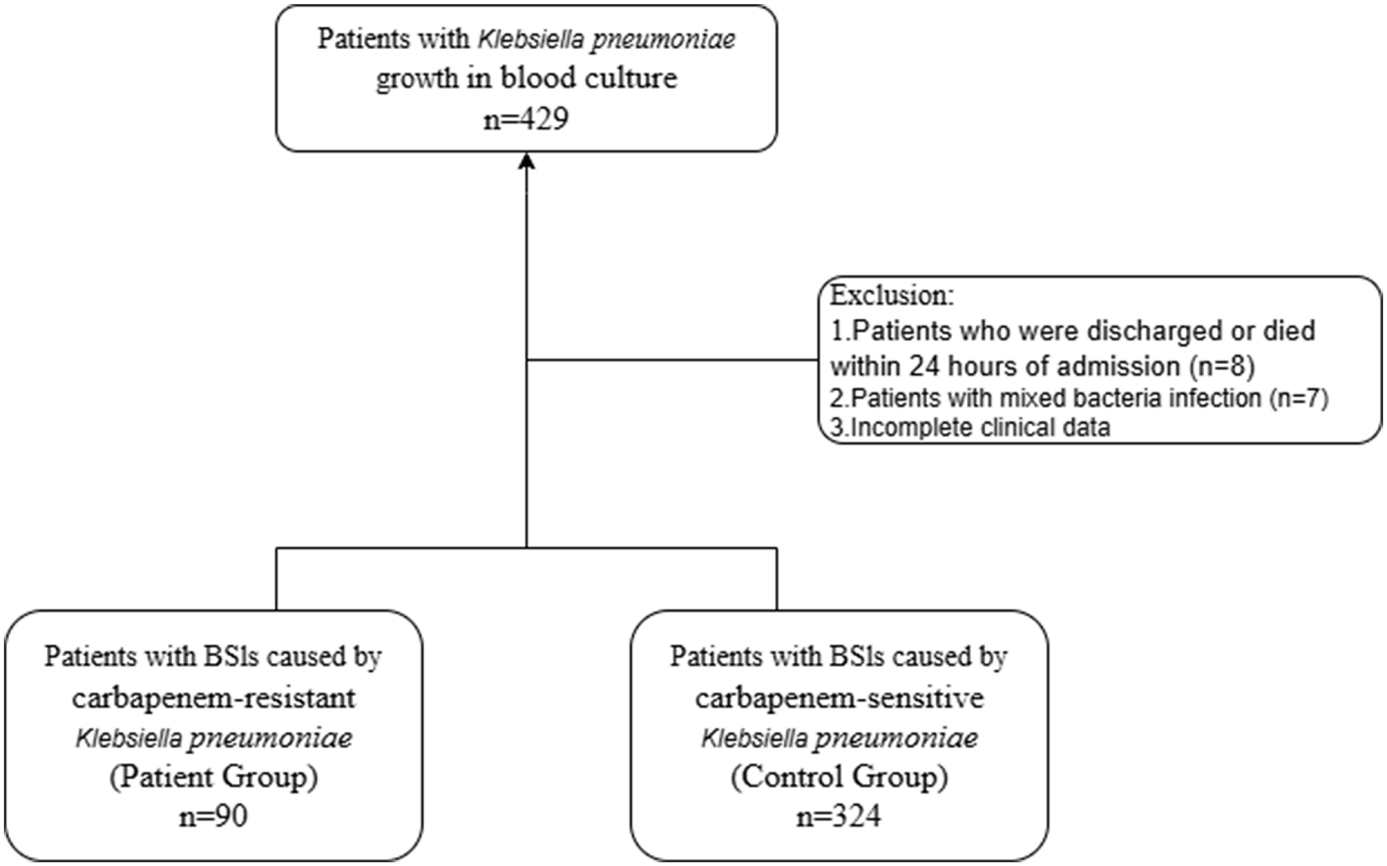
Study design showing study groups and excluded patients

The present study included patients with KP-BSI who were hospitalized in a tertiary hospital in Henan with 1,800 beds from January 1, 2019, to December 31, 2024. This was a retrospective cohort study. Data from 429 patients with KP-BSI were collected, and 414 patients were included. Based on bacterial resistance, patients were divided into CRKP-BSI and carbapenem-susceptible CSKP-BSI groups. The inclusion criteria were as follows: (1) *KP* was positive in blood culture at least once and the patient showed symptoms consistent with BSIs [9]; (2) age > 18 years; (3) presence of complete clinical data; and (4) record of only the first episode of BSI in the patient. The exclusion criteria were as follows: (1) discharge or death within 24 h of admission (*n* = 8); (2) multiple bacterial infections (*n* = 7); and (3) incomplete clinical data (Fig. 1).

### Bacterial identification and antimicrobial susceptibility testing

Blood cultures were performed using a BacT/Alert 3D automated blood culture system. Bacteria were identified, and their susceptibility to antimicrobials was assessed using the VITEK 2 Compact automated microbiological identification and susceptibility testing system. This was performed in accordance with the operating procedures of the instrument and reagent usage instructions. Additional susceptibility testing was conducted using the agar disc diffusion method on commercially prepared discs. The resulting data were interpreted according to the standards stipulated by the Clinical and Laboratory Standards Institute (CLSI M100-S33) [10]. Tigecycline susceptibility was assessed in accordance with the criteria stipulated by the US Food and Drug Administration (FDA) [11]. Colistin susceptibility was interpreted in compliance with the European Committee on Antimicrobial Susceptibility Testing (EUCAST) guidelines, as revised in 2019 [12]. The term CRKP was used to define *KP* isolates exhibiting resistance to ertapenem (MICs ≥ 2 µg/ml) or imipenem and/or meropenem (MICs ≥ 4 µg/ml). In the present study, CSKP was identified as a distinct category of CRKP isolates that demonstrated susceptibility to ertapenem, imipenem, and meropenem [13]. Quality control strains, comprising *Escherichia coli* ATCC 25922 and *Pseudomonas aeruginosa* ATCC 27853, were obtained from the National Center for Microbial Culture Collection.

### Data collection

A comprehensive dataset was extracted from each patient’s medical records, encompassing the following variables: demographic data (sex and age), age-adjusted Charlson comorbidity index (aCCI), underlying conditions (diabetes, hypertension, cardiovascular disease, cerebrovascular disease, solid tumors, and immunocompromised status), admission date, time of blood culture specimen submission, history of ICU admission, history of antibiotic exposure, history of previous surgeries, history of prior hospitalization, and discharge survival or death (in-hospital mortality). The primary aim of this study was to identify independent risk factors associated with the occurrence of CRKP-BSI. The secondary aim was to identify risk factors associated with in-hospital all-cause mortality. history of ICU admission was defined as admission to the intensive care unit at any time during the index hospitalization, history of antibiotic exposure was categorized based on predefined time intervals, including < 7 days, 7–30 days, 30–60 days, and 60–90 days before bloodstream infection, history of previous surgeries referred to surgical procedures performed within 30 days and within 30–90 days prior to infection onset, respectively, history of prior hospitalization was defined as any hospital admission within 12 months before infection. in-hospital all-cause mortality was defined as death from any cause that occurred during hospitalization in which KP-BSI were diagnosed. Nosocomial infections were identified based on the following criteria: absence of infection on hospital admission, onset after 48 h of hospitalization, and infection acquired during medical treatment in a healthcare setting. the primary infection source was defined as the initial site of infection from which the bloodstream infection was presumed to originate, based on clinical manifestations, microbiological evidence, imaging findings, and physicians’ assessments documented in the medical records.

### Statistical analysis

Data were analyzed using R statistical software (R Foundation; http://www.r-project.org; version 4.2.0) and EmpowerStats (www.empowerstats.net, X&Y Solutions, Inc., Boston, Massachusetts). Continuous variables with normal distributions are presented as the mean ± standard deviation (SD) when normally distributed, whereas those with non-normal distributions are reported as the median and interquartile range (IQR). Student’s t-test and the Mann–Whitney U test were used to compare normally and non-normally distributed continuous variables, respectively. Categorical variables were compared using either the chi-square test or Fisher’s exact test, depending on the suitability of the latter. Prior to the analysis, collinearity screening was performed. Univariate logistic regression analysis was conducted to assess the associations between the exposure variables. Significant variables (*P* < 0.05) from the univariate analysis were subsequently included in the multivariate regression model to identify independent risk factors for CRKP-BSI and in-hospital mortality. Odds ratios (OR) and 95% confidence intervals (CIs) were calculated for all variables. In multivariate logistic regression analysis, risk factors with *P* < 0.05 were considered statistically significant, with odds ratios (ORs) indicating the direction and strength of the association. An OR > 1 suggested an increased risk, whereas an OR < 1 indicated a potential protective effect.

## Results

### Antimicrobial susceptibility results for *Klebsiella pneumoniae*

The antimicrobial susceptibility profiles of *KP* bloodstream isolates revealed pronounced resistance to fluoroquinolones, with 45.9% resistance to ciprofloxacin. Among the cephalosporins, resistance to cefazolin was the highest (38.4%). Carbapenem resistance was substantial, at 21.7% for imipenem and 21.5% for meropenem.

Notably, several antimicrobial agents demonstrated promising efficacy against CRKP. Polymyxins exhibited exceptional activity, with an extremely low resistance rate of 0.5%. Ceftazidime-avibactam showed remarkable effectiveness, with only 1.7% resistance. Tigecycline maintained good activity, with an 8.7% resistance rate. Among the aminoglycosides, amikacin demonstrated superior activity to gentamicin, with resistance rates of 20.5% and 28.3%, respectively.

### Risk factors for CRKP-BSI

Compared with the CSKP-BSI group, the CRKP-BSI group presented considerably higher rates of multiple clinical characteristics, including ICU admission (24.4% vs. 7.4%, *P* < 0.001), healthcare-associated infection (85.6% vs. 32.1%, *P* < 0.001), hypertension (65.6% vs. 49.1%, *P* = 0.006), cerebrovascular disease (52.2% vs. 26.2%, *P* < 0.001), lower respiratory tract infection (64.4% vs. 31.5%, *P* < 0.001), and urinary tract infection (34.9% vs. 13.3%, *P* < 0.001) (Table 1).

**Table 1:** Comparison of clinical characteristics between patients with CRKP-BSIs and CSKP-BSIs

Variables	Total (*n* = 414)	CSKP (*n* = 324)	CRKP (*n* = 90)	*P*-value
Age, years	65.2 ± 14.8	65.8 ± 15.1	63.1 ± 13.5	0.14
BMI	23.7 ± 3.8	23.6 ± 3.9	24.0 ± 3.5	0.433
Sex (male)	275 (66.4%)	198 (61.1%)	77 (85.6%)	< 0.001
aCCI	4.0 (3.0–5.0)	4.0 ( 3.0–5.0)	3.0 (2.0–5.0)	0.121
ICU admission	46 (11.1%)	24 (7.4%)	22 (24.4%)	< 0.001
Hospital-acquired infection	181 (43.7%)	104 (32.1%)	77 (85.6%)	< 0.001
Hypertension	218(52.7%)	159 (49.1%)	59 (65.6%)	0.006
Coronary heart disease	82 (19.8%)	69 (21.3%)	13 (14.4%)	0.149
Diabetes mellitus	144 (34.8%)	123 (39.0%)	21 (23.3%)	0.01
COPD	7(1.7%)	5 (1.5%)	2 (2.2%)	0.649
Cerebrovascular disease	132 (31.9%)	85 (26.2%)	47 (52.2%)	< 0.001
Solid tumor	40 (9.7%)	33 (10.2%)	7 (7.8%)	0.494
Hematologic malignancy	37(8.9%)	34 (10.5%)	3 (3.3%)	0.035
Cardiac failure	24 (5.8%)	17 (5.2%)	7 (7.8%)	0.363
Liver diseases	25 (6.0%)	15 (5.7%)	3 (4.2%)	0.473
Biliary tract diseases	19 (4.6%)	16 (4.9%)	3(3.3%)	0.52
Previous hospitalization ^a^	115 (27.8%)	94 (29.0%)	21 (23.3%)	0.287
Renal diseases	40 (9.7%)	34 (10.5%)	6 (6.7%)	0.277
Autoimmune diseases	10 (2.9%)	9 (3.3%)	1 (1.3%)	0.352
Indwelling catheter	43 (10.4%)	21 (6.5%)	22 (24.4%)	< 0.001
Hemodialysis	18 (4.3%)	17 (5.2%)	1 (1.1%)	0.089
Previous use of antibiotics ^b^	19 (4.6%)	15 (4.6%)	4 (4.4%)	0.941
Previous use of antibiotics ^c^	41 (9.9%)	24 (7.4%)	17 (18.9%)	0.001
Previous use of antibiotics ^d^	60(14.5%)	40 (13.3%)	17 (18.9%)	0.181
Previous use of antibiotics ^e^	22 (5.3%)	17 (5.2%)	5 (5.6%)	0.908
SHOCK	33(8.0%)	27 (8.3%)	6 (6.7%)	0.606
Previous surgery ^f^	47 (11.4%)	16 (4.9%)	31 (34.4%)	< 0.001
Previous surgery ^h^	38 (11.0%)	23 (8.6%)	15 (19.7%)	0.006
Immunocompromised	43 (10.4%)	41 (12.7%)	2 (2.2%)	0.004
Central venous catheterization	109 (26.3%)	71 (21.9%)	38 (42.2%)	< 0.001
Primary infection sources				
Lower respiratory tract	160 (38.7%)	102 (31.5%)	58 (64.4%)	< 0.001
Abdominal cavity	53 (12.8%)	47 (14.5%)	6 (6.7%)	0.049
Urinary tract	125 (30.2%)	113 (34.9%)	12 (13.3%)	< 0.001
Other	76 (18.4%)	62 (19.1%)	14 (15.6%)	0.438

Notes: Data are expressed as number (%), mean ± SD, or median (IQR)

Abbreviations: BMI, body mass index; ICU, intensive care unit; COPD, chronic obstructive pulmonary disease; aCCI, age-adjusted Charlson Comorbidity Index

^a^ During the 12 months preceding infection onset, ^b^ During the 60–90 days preceding infection onset, ^c^ During the 30–60 days preceding infection onset, ^d^ During the 7–30 days preceding infection onset. ^e^ During the <7days preceding infection onset. ^f^ During the 30 days preceding infection onset. ^h^ During the 30–90 days preceding infection onset

The findings from the multivariate analysis indicated that independent risk factors for the occurrence of CRKP-BSIs included ICU admission (OR = 10.49, *P* = 0.0002), hospital-acquired infections (OR = 9.34, *P* < 0.0001), hypertension (OR = 2.85, *P* = 0.0106), and surgical procedure within 30 days (OR = 5.88, *P* = 0.0002). Notably, female sex was associated with a significantly reduced risk of developing CRKP-BSIs (OR = 0.18, *P* = 0.0006) (Table 2).

**Table 2:** Multivariate analysis of factors associated with the development of CRKP-BSI

Variable	OR	(95% CI )	*P*-value
Sex (female)	0.18	0.07–0.49	0.0006
Hypertension	2.85	1.28–6.37	0.0106
Diabetes mellitus	0.63	0.26–1.50	0.2933
Cerebrovascular disease	0.74	0.32–1.72	0.4812
Hematologic malignancy	0.75	0.13–4.38	0.7479
Immunocompromised status	0.21	0.03–1.43	0.1100
aCCI > 3	0.67	0.31–1.48	0.3222
ICU admission	10.49	3.02–36.51	0.0002
Hospital-acquired infection	9.34	3.94–22.13	< 0.0001
Indwelling catheter	2.62	0.88–7.74	0.0820
Central venous catheterization	1.56	0.69–3.53	0.2899
Previous antibiotic use ^a^	2.05	0.75–5.58	0.1602
Surgical procedure ^b^	5.88	2.31-15.00	0.0002
Surgical procedure ^c^	0.76	0.26–2.26	0.6228
Primary Infection source			
Lower respiratory tract	1.86	0.85–4.07	0.1182
Abdominal cavity	0.56	0.12–2.63	0.4672
Urinary tract	0.63	0.18–2.23	0.478

Abbreviations: ICU, intensive care unit; aCCI, age-adjusted Charlson Comorbidity Index

^a^ During the 60 days preceding infection onset; ^b^ During the 30 days preceding infection onset; ^c^ During the 90 days preceding infection onset

### Analysis of risk factors for in-hospital mortality in patients with KP-BSI

The analysis of the risk factors for inpatient mortality associated with KP-BSIs is presented in Table 3. The overall in-hospital mortality rate was 11.8%. The mortality rates of patients with CRKP-BSI and CSKP-BSI were 21.1% and 9.3%, respectively. Univariate analysis revealed that ICU stay, lower respiratory tract infection, urinary tract infection, shock, central venous catheterization, and CRKP infection were associated with increased inpatient mortality. Multivariate analysis revealed that ICU admission (OR = 6.03, *P* < 0.0001) and lower respiratory tract infection (OR = 2.44, *P* = 0.0103) were independent risk factors for inpatient death.

**Table 3:** Association between in-hospital mortality and potential independent variables: results of univariate and multivariate analysis

Risk Factor	Univariate analysis			Multivariate analysis	
	Survivors (*n* = 365)	Death (*N* = 49)	*P*-value	OR (95%CI)	*P*-value
Sex (male) n (%)	244 (66.8)	31 (63.3)	0.6182		
aCCI >3	212 (58.1)	34 (69.4)	0.1330		
ICU admission	27 (7.4)	19 (38.8)	< 0.0001	6.03 (2.57–14.13)	<0.0001
Hospital-acquired infection	155 (42.5)	26 (53.1)	0.1625		
Hypertension	193 (52.9)	25 (51)	0.8070		
Coronary heart disease	72 (19.7)	10 (20.4)	0.9104		
Diabetes mellitus	130 (35.6)	14 (28.6)	0.3325		
COPD	6 (1.6)	1 (2.0)	0.8399		
Cerebrovascular disease	113 (31)	19 (38.8)	0.2719		
Solid tumor	38 (10.4)	2 (4.1)	0.1758		
Hematologic malignancy	33 (9)	4 (8.2)	0.8398		
Cardiac failure	20 (5.5)	4 (8.2)	0.4535		
Liver diseases	23 (6.3)	2 (4.1)	0.5435		
Biliary tract diseases	17 (4.7)	2 (4.1)	0.8566		
Previous hospitalization ^a^	104 (28.5)	11 (22.4)	0.3766		
Renal diseases	35 (9.6)	5 (10.2)	0.8912		
Autoimmune diseases	9 (2.5)	1 (2.0)	0.9875		
Indwelling catheter	37 (10.1)	6 (12.2)	0.6503		
Hemodialysis	14 (3.8)	4 (8.2)	0.1734		
Previous use of antibiotics ^b^	16 (4.4)	3 (6.1)	0.5867		
Previous use of antibiotics ^c^	34 (9.3)	7 (14.3)	0.2781		
Previous use of antibiotics ^d^	53 (14.5)	7 (14.3)	0.9650		
Previous use of antibiotics ^e^	18 (4.9)	4 (8.2)	0.3490		
SHOCK	25 (6.8)	8 (16.3)	0.0261	1.36 (0.47,3.94)	0.5766
Previous surgery ^f^	42 (11.5)	5 (10.2)	0.7874		
Previous surgery ^b^	34 (9.3)	4 (8.2)	0.8830		
Immunocompromised	38 (10.4)	5 (10.2)	0.9645		
Central venous catheterization	90 (24.7)	19 (38.8)	0.0375	0.84 (0.40,1.77)	0.6542
CRKP infection	71 (19.5)	19 (38.8)	0.0027	1.44 (0.69,2.99)	0.3260
Primary Infection source					
Lower respiratory tract	129 (35.3)	31 (63.3)	0.0003	2.44 (1.23,4.83)	0.0103
Abdominal cavity	48(13.2)	5(10.2)	0.5633		
Urinary tract	119(32.6)	6(12.2)	0.0057	0.69 (0.24, 1.97)	0.4884
Other	69(18.9)	7(14.3)	0.4347		

Notes: Data are expressed as number (%) or mean ± SD or median (IQR)

Abbreviations: ICU, intensive care unit; COPD, chronic obstructive pulmonary disease; aCCI, age-adjusted Charlson Comorbidity Index

^a^ During the 12 months preceding infection onset, ^b^ During the 90 days preceding infection onset, ^c^ During the 60 days preceding infection onset, ^d^ During the 30 days preceding infection onset. ^e^ During the 7–30 days preceding infection onset. ^f^ During the 30 days preceding infection onset

## Discussion

In this six-year retrospective cohort study, we systematically compared carbapenem-resistant and carbapenem-susceptible Klebsiella pneumoniae bloodstream infections and identified factors associated with both CRKP-BSI occurrence and in-hospital mortality. We found that ICU admission during hospitalization, hospital-acquired infection, hypertension, and recent surgical intervention were independently associated with the development of CRKP-BSI. In addition, ICU admission and lower respiratory tract infection were identified as independent risk factors for in-hospital mortality among patients with KP-BSI. These findings are largely consistent with previous reports, while providing region-specific evidence from a high-burden area in central China.

In recent years, *KP resistance* to carbapenems has increased [4, 14]. In the present study, *KP* resistance rates to imipenem and meropenem were 21.7% and 21.5%, respectively. These rates are lower than the CRKP resistance rates of 37.3% and 37.7% reported in previous single-center studies from Hebei Province in Northern China and Anhui Province in Southern China, respectively [15, 16]. This discrepancy may be attributed to regional epidemiological factors, prescription habits, and infection control measures. Notably, high resistance rates were observed for ciprofloxacin, tetracycline, and cefazolin, suggesting that these traditional antibiotics have limited efficacy in treating *KP* infections and should be used based on susceptibility testing. *KP* was highly susceptible to tigecycline and amikacin during this period, indicating their strong antibacterial activity. Furthermore, polymyxin and ceftazidime-avibactam exhibited the lowest resistance rates, suggesting their substantial in vitro antibacterial activity. These findings indicate that polymyxin and ceftazidime-avibactam are effective treatment options for drug-resistant *KP* infections. However, it is important to note that polymyxins are frequently used as part of combination therapy, which generally refers to the concomitant intravenous administration of polymyxins with another active Gram-negative agent to enhance antibacterial efficacy and reduce the risk of resistance emergence [17]. Ceftazidime-avibactam demonstrates effective antibacterial activity against *KP* strains that produce KPC enzymes; however, it is ineffective against strains that produce metallo-beta-lactamases. Consequently, it is imperative to obtain clear susceptibility results when these antibiotics are used, and carbapenemase typing may be required. The Infectious Diseases Society of America and European Society of Clinical Microbiology and Infectious Diseases guidelines recommend ceftazidime-avibactam as a first-line treatment option for CRKP infections [18, 19].

Our analysis of CRKP-BSI risk factors is largely consistent with findings from previous meta-analyses and cohort studies, while also providing region-specific evidence from a high-burden setting [20]. In our study, ICU admission during hospitalization was strongly associated with the development of CRKP bloodstream infection, with patients admitted to the ICU showing a markedly higher risk of CRKP-BSI. This finding aligns with prior reports demonstrating that critically ill patients are particularly vulnerable to CRKP infection due to frequent exposure to invasive procedures, severe underlying illness, and extensive use of broad-spectrum antibiotics [8, 21]. These results highlight the importance of heightened surveillance and strict infection control practices in ICU settings. In addition, surgical intervention within 30 days prior to infection was identified as an independent risk factor for CRKP-BSI in our cohort. Similar associations between recent surgical procedures and CRKP infection have been reported in previous studies [8, 16]. Surgical interventions may increase susceptibility to CRKP-BSI by disrupting anatomical barriers, prolonging hospitalization, and increasing the need for postoperative invasive devices and antimicrobial exposure, which has been associated with an increased risk of carbapenem-resistant organism colonization and infection [16, 22].

In our cohort, 85.6% of CRKP cases were hospital-acquired infections, consistent with related epidemiological reports [22]. This high proportion highlights the risk of nosocomial transmission and the need for robust infection-control strategies. Hypertension has emerged as a significant high-risk factor, likely due to the elevated risk of stroke in patients with high blood pressure [23]. Patients who have experienced a stroke may have impaired consciousness, consequently increasing their susceptibility to secondary pulmonary infections or necessitating ICU admission after surgical interventions. Furthermore, our study has identified female sex as an independent protective factor against CRKP-BSI. This result consistent with previous studies that have reported either a higher incidence in males [24] or a non-significant association following multivariate adjustment [25]. These discrepancies underscore the possibility that the role of sex may be influenced by population-specific factors, such as regional epidemiology and local clinical practices. Consequently, our findings emphasize the importance of considering demographic context in risk assessment and advocate for future research to elucidate the specific mechanisms—whether biological or behavioral—that confer this protective effect.

The observed discrepancy between univariate and multivariate analyses regarding Antibiotics administered 60 days prior to infection is not unexpected. and has been reported in previous studies of carbapenem-resistant Enterobacterales. Antibiotic exposure is often closely correlated with other clinical factors, such as ICU admission, severity of illness, invasive procedures, and prolonged hospitalization, which may attenuate its independent effect after multivariable adjustment [8, 16, 20]. In addition, the impact of prior antibiotic exposure on the emergence of drug-resistant organisms may be time-dependent, with more recent exposure exerting a stronger selective pressure than antibiotic use occurring further from the onset of infection. Differences in antibiotic classes, duration of therapy, and cumulative exposure may also contribute to the heterogeneous effects observed across studies. Taken together, these findings highlight the complex and context-dependent relationship between prior antibiotic exposure and the risk of CRKP bloodstream infection.

These findings not only contribute to our understanding of CRKP-BSI risk factors but also highlight critical areas for future investigation. The observed variations, particularly in sex-specific infection risk and antibiotic exposure patterns, suggest the need for larger multicenter prospective studies to elucidate the underlying mechanisms. Our findings reinforce existing evidence regarding risk factors for CRKP bloodstream infection and provide additional region-specific data that may help identify patients at higher risk in clinical practice.

In this study, the in-hospital mortality rate for patients with KP-BSI was 11.8%, with the mortality rate for CRKP-BSI being higher than that for CSKP-BSI (21.1% vs. 9.3%). This rate appears lower than those reported in previous studies, which have documented in-hospital mortality rates ranging from 30% to 60% for patients with CRKP-BSI [8, 15, 16, 20]. Several factors may contribute to this difference, including variations in patient populations, infection severity, antimicrobial management, and institutional infection control practices. Additionally, the lower mortality in our cohort may be partly due to the transfer of critically ill patients to higher-level hospitals; however, data on patient transfers and follow-up were not available.

Subsequent analysis of the risk factors for in-hospital mortality revealed that a history of ICU admission and lower respiratory tract infections were independent risk factors. ICU admission is a significant risk factor for CRKP infection. This association is likely mediated by multiple factors, including invasive procedures, antibiotic exposure, and patient vulnerability, which collectively contribute to an increased infection risk and potentially higher mortality. This finding is consistent with those of previous studies [8, 26] that emphasized the vulnerability of ICU patients to severe infections. Lower respiratory tract infections have been identified as the primary source of KP-BSI, with the potential to markedly increase the risk of mortality by directly inducing septicemia or by interacting with factors such as mechanical ventilation and resistance. This finding is consistent with the results of previous studies [7] that highlighted the critical role of lower respiratory tract infections in increasing the mortality risk of bloodstream infections. These risk factors underscore the necessity for enhanced infection control measures in patients admitted to the ICU and those with respiratory infections.

### Limitations of this study

The present study had several limitations. First, this was a single-center retrospective study, which may have introduced selection bias and inadequacy in controlling for confounding factors. Second, the antimicrobial susceptibility test panel used in this study did not include ertapenem. Consequently, although ertapenem is part of the phenotypic definition of CRKP, we were unable to determine its resistance rate. Identification of CRKP was primarily based on resistance to imipenem and/or meropenem. Third, the retrospective nature of this study limited the availability of detailed antibiotic use data. Pre-admission antibiotic exposure information was often incomplete owing to inconsistent documentation of treatments initiated in primary care settings. Additionally, a detailed analysis of in-hospital antimicrobial therapy was not feasible because of the complex, dynamically adjusted nature of the treatment regimens based on the clinical course and microbiological results. Future prospective studies are warranted to comprehensively evaluate the impact of antibiotic stewardship on patient outcomes. Fourth, this study was limited by the lack of molecular data on carbapenemase genes. As this was a retrospective analysis reflecting routine clinical practice, carbapenemase detection was not performed because phenotypic susceptibility testing remains the standard for clinical decision-making at our institution. Future prospective studies incorporating genotypic characterization should provide valuable insights into the specific resistance mechanisms circulating in the region. Finally, the mortality analysis conducted in this study was confined to in-hospital outcomes and utilized logistic regression. Although this method is effective in identifying significant risk factors, it fails to consider time-to-event dynamics. Future prospective studies that include post-discharge follow-up should employ survival analysis techniques, such as Cox proportional hazards regression, to offer a more comprehensive understanding of mortality risk over time.

## Conclusion

This study highlights the substantial epidemiological distinctions between CRKP-BSI and CSKP-BSI. The incidence of CRKP-BSI is influenced by several factors, including ICU admission history, nosocomial infections, hypertension, and surgery within 30 days. Furthermore, ICU admission and lower respiratory tract infections were identified as independent risk factors for in-hospital mortality in patients with KP-BSI. These findings emphasize the importance of targeted infection control strategies and rational antibiotic use to reduce the incidence of KP-BSI and improve patient outcomes.

## Data Availability

The data sets generated during and/or analysed during the current study are available from corresponding author on reasonable request.

## Abbreviations

BMI: Body mass index
ICU: Intensive care unit
COPD: Chronic obstructive pulmonary disease
aCCI: Age-adjusted Charlson Comorbidity Index
OR: Odds ratios
CRKP: Carbapenem-resistant *Klebsiella pneumonia*
BSIs: Bloodstream infections
